# Does nonreproductive swarming adapt to pathogens?

**DOI:** 10.1371/journal.ppat.1006742

**Published:** 2018-01-25

**Authors:** Qingyun Diao, Chunsheng Hou

**Affiliations:** 1 Institute of Apicultural Research, Chinese Academy of Agricultural Sciences, Haidian District, Beijing, P. R. China; 2 Key Laboratory of Pollinating Insect Biology, Ministry of Agriculture, Haidian District, Beijing, P. R. China; Geisel School of Medicine at Dartmouth, UNITED STATES

## What is reproductive and nonreproductive swarming in honey bees?

Honey bees play a vital role in the pollination of crops, fruits, and wild plants [1]. However, pathogens and parasites pose major threats to their fitness and survival due to the large number of declines reported recently in North America and several European countries [2]. Aside from the innate immunity of honey bees, indirect fitness benefits resulting from altered behavior may provide resistance against pathogens and parasites [3]. Although the study of vertebrate behavior has received increasing attention from researchers [4], the role of behavior in insect immunity has been much less appreciated.

A recent publication regarding the incidence of pathogens in honey bee colonies and honey bee swarming behavior has positioned the honey bee at the forefront of efforts to understand the relationship between pathogens and complex behaviors [5] such as warning their nestmates of danger from predators [6]. Honey bee swarming, also termed reproductive swarming, is a natural division of the hive population and colony fission; when the number of workers exceeds the capacity of the hive, the old queen will travel to a new location with approximately three-fourths of the colony’s worker bees and drones. Generally, this swarming occurs between 10 AM and 2 PM on a warm sunny day in the spring or summer, seasons with sufficient plant nectar and temperate climate to facilitate the development and growth of the new colony [7]. Due to rapid growth, reproductive swarming is also useful for increasing the population and maintaining a high level of reproduction during times of rich food resources, and it is the major way of reproduction of honey bee colonies [8].

By contrast, however, nonreproductive swarming does not typically occur in spring or summer but in seasons in which there is insufficient plant nectar or when the hive has enough space to accommodate more honey bees. Nonreproductive swarming is mainly a resistance mechanism at the colony level [9]. Compared to reproductive swarming, incidents of nonreproductive swarming of honey bee colonies were found to be increased after July, which is not a reproductive season (S1 and S2 Movies). Therefore, these findings demonstrated that nonreproductive swarming as a collective behavior might have an additive function for colony survival or health.

More importantly, honey bees cannot reproduce or ensure healthy hive development if they are unable to ensure their survival first [10]. Within this context, the relevance between nonreproductive swarming and population health is especially important. This article will focus on the relationship between nonreproductive swarming and pathogens and try to understand the possible functions of this collective behavior.

## More nonreproductive swarming found in colonies with more pathogens

One of the most effective mechanisms by which honey bees can protect themselves against pathogens or parasites is by changing their behavior [11]. Honey bees are a social insect, and they rely mainly on behavioral and physiological defenses against diseases [7]. Although habitat loss [12] and pesticides [13] have been confirmed to impair honey bee health, pathogens are considered the most serious threat [14]. Microbial pathogens are thought to have a profound impact on insect populations [15]. For example, the combination of pathogen stress with other stressors can induce the decline of a honey bee colony or even entire colony collapse [2]. Therefore, when the physiological defenses of the individual are inadequate, colony survival requires collective behaviors to eradicate pathogens or parasites [3].

**Fig 1:**
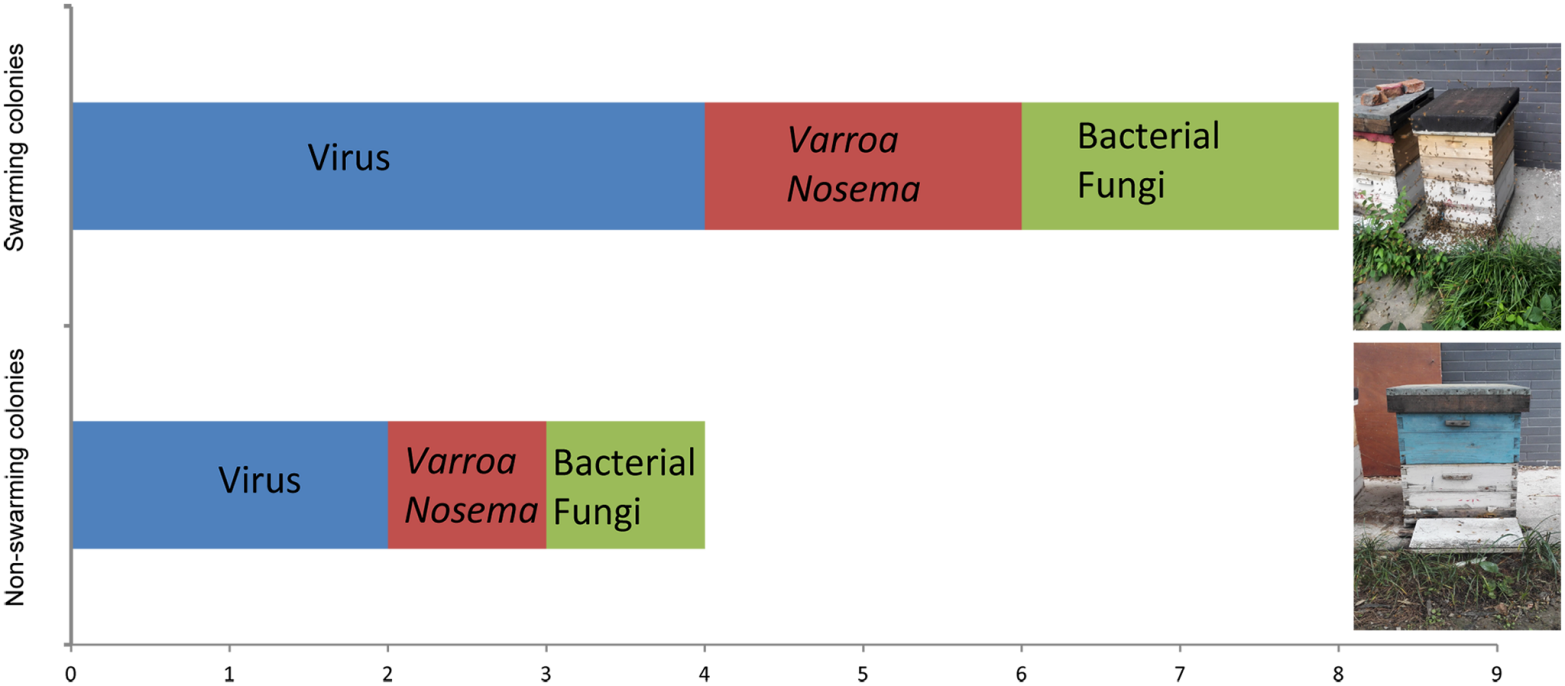
The benefits of nonreproductive swarming. Nonreproductive swarming occurs more often in colonies with more pathogens (above) than in colonies with fewer pathogens (below) when other conditions are comparable [2,5,9,16,17]. The 1–9 scale represents the average number of pathogens or parasites in the colonies. *Varroa* and *Nosema*, and bacteria and fungi, indicate that at least one of them will be found in swarming or nonswarming colonies.

Reproductive swarming generally occurs between 5 May and 21 July in years when resources to support rapid population growth are plentiful and typically occurs fewer than three times in one colony per year [16]. As shown in S1 and S2 Movies, however, it was found that swarming has not been occurring at the typical times, and this nonreproductive swarming occurs mostly in colonies for which conditions become unfavorable to defense against pathogens [9]. Moreover, nonreproductive swarming due to the influence of pathogens is increased, even in honey bee colonies maintained under the same conditions when the number of pathogens have to reach a certain threshold (Fig 1) [2,5,9,16,17]. Additionally, honey bee virus titers are usually elevated in the autumn or winter months, and the intensity of Deformed Wing Virus (DWV) infection increases during this period, causing colony decline [17]. Loftus et al. [5] found that colonies in small hives swarmed more often, had lower *Varroa* infestation rates, less disease, and had higher survival rates compared with colonies in large hives. In addition, it was found that the infection percentages of DWV (58%) and Sacbrood Virus (SBV; 66.7%) were higher in large hive colonies than in small hive colonies (0%). Similarly, not only does casting a swarm export approximately 35% of a colony’s *Varroa*—because approximately 70% of the adult bees leave when a colony casts a swarm [18], and approximately 50% of the *Varroa* in a colony are on the adult bees—it also creates a broodless period in the swarming colony [19]. This broodless period may help further shrink the *Varroa* population in a colony that has swarmed because *Varroa* depends on honey bee broods for reproduction. In addition, by swarming, honey bees (*Apis florea*) migrate to new environments for survival when they are under threat from a predator [20].

## Is nonreproductive swarming one form of behavioral immunity?

Behavioral variation often results from differences in response thresholds to key sensory stimuli. Much research has focused on understanding the genetic and physiological immunity against parasites, but hosts can also use behaviors to avoid infection, reduce parasite growth, or alleviate disease symptoms [3]. Insects can reduce their risk of exposure to parasites by avoiding an infectious habitat, either through migration or swarming [3]. Long-term migration also reduces parasite prevalence by weeding out infected individuals [21]. For example, through migration and swarming, honey bees can find new sources of nectar and hive locations, but migration is generally used to obtain sufficient floral resources [4]. Honey bees, therefore, have to employ swarming to avoid pathogens, meaning that nonreproductive swarming may occur during seasons with fewer flower resources to allow the honey bees to avoid pathogens and parasites [5,9].

Host adaptations to novel environmental conditions can favor some level of reproductive isolation and may ultimately lead to ecological speciation and adaptive radiation in insects [3]. Spatial avoidance of parasites is especially prevalent in the context of female oviposition behavior. Water striders (*Aquarius paluduminsularis*), for example, tend to oviposit deep underwater to avoid infection of the egg with the parasitoid wasp *Tiphodytes gerriphagus* [22]. Butterflies also abandon parasite-infested host plant habitats and recolonize parasite-free habitats [3]. Many female insects crucially and directly affect the fitness of their offspring by determining where they lay their eggs, and there should be strong selection for females that lay their eggs in habitats that result in high offspring performance [23].

Contagious sexually transmitted diseases are common in natural populations, and hosts should strongly prefer healthy uninfected partners to avoid infection during mating [24]. Fitness benefits can be gained when the preference for a healthy mate is associated with heritable resistance to and/or tolerance for parasites that will be passed to offspring [25]. Therefore, nonreproductive swarming can reduce the risk of infection by actively avoiding infected sexual partners, or habitat choice to escape parasites can result in niche expansion of insect hosts due to honey bee mating in the air [26].

## Nonreproductive swarming might be one collective behavior that is involved in pathogen resistance

Nonreproductive swarming might be a honey bee collective behavior that eradicates pathogens from the colony. Honey bees lack adaptive immunity and many canonical immune genes that other insects possess, and behavioral evolution is therefore an alternative defense mechanism allowing honey bees to respond to environmental changes [27]. Behavioral changes of social insects that result from an infection can reflect either a successful host manipulation by the pathogen or an adaptive response by the host [28]. Kin selection has repeatedly led to the evolution of complex insect societies that are characterized by various forms of altruistic behavior [29]. Disease transmission in colonies is facilitated by the close physical contact and social feeding of nestmates (trophallaxis). Self-removal and suicide of diseased or parasitized individuals may thus decrease the infection risk for surrounding kin [30]. Ant workers will cease social contact and leave the colony if they are infected with an entomopathogenic fungus [29]. Likewise, exposure of honey bees to CO_2_ narcosis or hydroxyurea induces the surviving foragers to neglect their social functions and remove the diseased individual from the colony; these conditions also lead to altruistic suicide, in which developmentally deformed workers crawl out of the hive [29]. Similarly, a recent study confirmed that workers from swarming colonies have high reproductive potential and live longer than those from the original colony [31]. Therefore, movement and habitat use may be strong determinants of honey bee avoidance of pathogen and parasite transmission [3]. As investigated by Vardayani et al. [8], swarming significantly reduced disease infestation with *Varroa* and Acute Bee Paralysis Virus in the parent colony. Although hygienic behavior of honey bees has been considered an important immune response of pathogen resistance, left beehives were very common following an outbreak of American foulbrood, which resulted in a heavy cost due to the removal of healthy larvae [32].

## Nonreproductive swarming can increase genetic diversity

Genetic diversity is critical to ensuring the fitness of populations. Swarming naturally selects for population reproduction and development and is a mechanism for increasing genetic diversity because a new queen (swarming will produce a new queen) can mate with more than one drone [33]. Genetic diversity in individuals related to foraging rates, food storage, and population growth led to boosts in fitness of the individuals, and when circumstances change, an organism’s first response is often behavioral, as in the evolution in adaptive behaviors. Because genetic diversity in a honey bee colony enhances its productivity and health, producing swarming is one of the ways to propagate its gene [33]. As indicated by Clive et al. [34], mating is associated with increased survival and breeding in the next season. A genetically diverse host population is less likely to provide a favorable environment for pathogens, significantly reducing the prevalence and incidence of diseases; similar to how highly diverse plants have lower infection rates than less diverse plant species [35]. Evidence has shown that polyandry allows species to better resist infection by pathogens [36].

Through losses of genetic diversity, the prevalence of the gut parasite *Crithidia bumbi* was higher in lower genetic diversity populations, and the level of phenoloxidase was negatively correlated with parasite abundance in bumblebees [37]. The swarming colony mostly consisted of the older bees, which could explain the colony’s drive to increase its genetic diversity and avoid inbreeding [38].

Swarming is a necessary and sufficient behavior of honey bees used as a problem-solving system that allows them to self-organize and adapt [39]. Desai et al. showed that, compared to genetically homogeneous colonies, genetic diversity provides increased resistance to multiple pathogens and to the parasitic mite *Varroa*. They found that colonies inseminated by multiple drones and thus having greater within-colony genetic diversity showed reduced prevalence and pathogen concentrations of the parasites *Nosema* and *Varroa* and of seven honey bee viruses [40]. These findings suggest that genetically diverse honey bee populations can recover from introduced diseases by evolving rapid tolerance or resistance while maintaining much of their existing genetic variation [41].

## Concluding remarks

Swarming studies have taught us that a series of conditions must be met to determine whether nonreproductive swarming is an antiparasitic defense or is for better survival and development. We must therefore ask if this behavior alleviates fitness loss. Although the study of behavioral immunity in insects is still in its infancy, it is already clear that insects display an enormous variety of antiparasitic behaviors [3]. Like grooming behaviors, nonreproductive swarming may serve multiple functions for population survival and growth [3,9].

Overall, although defensive behavior in honey bee colonies is a complex system influenced by many interacting environmental and genetic factors [42], recent progress in understanding swarming and colony health have helped us learn more about the effects of honey bee behavior on the resistance to pathogen infection [5,9,31]. Finally, it is worth mentioning that honey bees may use nonreproductive swarming as a possible strategy to separate pathogens from the colony, but how this population behavior has evolved to counteract pathogen attacks remains to be determined. We hope that this review will inspire researchers to consider the exciting behaviors that may have evolved in response to parasites in honey bees. In addition, we will further supply more experimental evidence to confirm how these findings are translated at the genomic and physiological levels into changes in behavior. Exploring population behavior could lead to novel management and genetic control strategies for improving the resistance of honey bees against important pathogens or parasites.

## Supporting information

S1 Movie The nonreproductive swarming was recorded on 29 August 2015 in Liaoning province. (MP4)

S2 Movie The nonreproductive swarming was recorded on 13 September 2016 in Beijing province. (MP4)
